# Impact of clinical pharmacist collaboration in patients beginning insulin pump therapy: a retrospective and cross-sectional analysis*

**DOI:** 10.3109/21556660.2013.815624

**Published:** 2013-06-19

**Authors:** James L. Ledford, Rick Hess, Frank P. Johnson

**Affiliations:** 1 East Tennessee State University, Bill Gatton College of Pharmacy Johnson City, TNUSA; 2 State of Franklin Healthcare Associates, Johnson City Internal Medicine Associates Johnson City, TNUSA

**Keywords:** Continuous Subcutaneous Insulin Infusion (CSII), Pharmacist, Diabetes, Primary care, Interprofessional collaboration

## Abstract

**Objective:**

To measure clinical and qualitative outcomes in patients with diabetes mellitus transitioning from intensive insulin therapy using multiple daily injections (MDI) to continuous subcutaneous insulin infusion (CSII) initiated and managed by clinical pharmacists under a collaborative practice agreement in a primary care setting without an endocrinologist.

**Research design and methods:**

This study was a retrospective and cross-sectional analysis of data from an electronic medical record (EMR) and patient survey at a large primary care private practice. Patients with type 1 or type 2 diabetes who were ≥18 years old, started on CSII between 2007 and 2010, and had at least one follow-up visit post-CSII were analyzed. Mean HbA1c results were stratified across 3-month intervals post-CSII initiation and compared to pre-CSII levels. Body mass index (BMI), the number of diabetes-related clinic visits with the primary care physician (PCP), and non-insulin diabetes medication use was compared pre- and post-CSII initiation. Paper-based questionnaires were used to assess patient satisfaction with CSII vs MDI and pharmacist-led services.

**Results:**

Twenty-five patients were included in the analysis. HbA1c decreased from 8.69 to 7.52% pre and post-CSII, respectively (*p* < 0.001). HbA1c also decreased across all 3-month intervals post-CSII. BMI decreased from 33.0 to 32.3 kg/m^2^ pre- and post-CSII, respectively (*p* = 0.085). Fewer diabetes-related PCP visits were completed post-CSII (5.09 vs 3.78 visits/year, *p* = 0.009), and less non-insulin diabetes medications were prescribed post-CSII (*p* < 0.001). Patients felt more comfortable controlling glycemic excursions and resultant insulin adjustments with CSII compared to MDI (*p* < 0.001).

**Conclusions:**

Pharmacist-led CSII services appear to improve diabetes control in patients requiring intensive insulin therapy. Patients report greater comfort using CSII and strong confidence in the abilities of the pharmacist. Physician–pharmacist collaboration in the management of intensive insulin therapy in the primary care setting should be further explored.

## Introduction

Diabetes is a chronic metabolic disease with the potential for several complications requiring close medical management in primary care settings. The Centers for Disease Control and Prevention (CDC) estimates that the prevalence of diabetes in the US is at a record high, with ∼18.8 million diagnosed and 7 million undiagnosed individuals with diabetes1. Insulin therapy delivered via multiple daily injections (MDI), also known as basal-bolus therapy, or by continuous subcutaneous insulin infusion (CSII) is a mainstay of treatment for type 1 diabetes upon diagnosis. Type 2 disease, the most common form treated in primary care, is progressive and aggressive insulin intensification to safely control diabetes may be required over time.

CSII, also known as insulin pump therapy, is an alternative method of insulin delivery for patients requiring intensive therapy. Studies indicate CSII lowers HbA1c and improves the quality-of-life in patients with diabetes2–9. However, the conversion from MDI to CSII is not a simple transition requiring only one 15 min office visit. Appropriate training for patients converting to this new approach can be a time-consuming process and requires clinical personnel with expertise on all facets of insulin pump therapy. Therefore, health professionals trained in CSII-based therapy are able to collaborate with primary care providers in order to provide this specialized service of diabetes care. This potential relationship is important because, as the need for primary care practitioners (PCPs) increases, the influx of new PCPs is decreasing10. Not only is there a shortage of PCPs, there is also a large, unsatisfied demand for endocrinologists due to the expected rise in the number of patients with diabetes and other endocrine disorders11. As a result, opportunities for interprofessional collaboration with other healthcare providers, such as pharmacists, are available to provide diabetes clinical services and education in a primary care setting.

Pharmacists are positioned to contribute to patient care outside of their traditional roles (i.e., medication dispensing) as the population with chronic diseases steadily grows. A US Public Health Service report published recently advocates for more pharmacist-delivered care due to an increase in chronic diseases, decreased access to care, and a declining primary care workforce12. Physician–pharmacist collaboration in primary care settings has demonstrated improved outcomes in chronic disease management12–18. This is especially true in the management of patients with diabetes. The addition of pharmacists to primary care teams has reduced cardiovascular risk14, and improved blood pressure control15, and cholesterol management16 in patients with diabetes. In addition, adding pharmacists to primary care teams has improved guideline-adherent use of anti-platelet medications17, as well as improved the cost-effectiveness of providing care18. Physician–Pharmacist collaboration efforts centered on insulin initiation in veterans with uncontrolled type 2 diabetes has been previously described19 and intensification of anti-diabetes therapies, including insulin, has demonstrated a reduction in HbA1c20,21. Only one CSII-based study to date included clinical pharmacists working in collaboration with a multidisciplinary team with access to endocrinologists3. The current study involved pharmacists operating under collaboration with PCPs only, which more accurately represents the majority of primary care practice settings today.

Diabetes medical management is becoming increasingly complex as new therapeutic agents come to market. At the same time, diabetes-related technology is evolving with medical devices such as insulin pumps offering several alternatives to physicians and patients. The formal training of medications and medication-related devices position pharmacists as collaborating partners in the care for patients with diabetes. The primary objective of this study was to measure clinical outcomes in patients with diabetes mellitus transitioning from intensive insulin therapy with MDI to CSII initiated and managed by clinical pharmacists under a collaborative practice agreement in a primary care setting without an endocrinologist. The secondary objective of this study was to assess patient satisfaction with CSII compared to MDI and with the clinical pharmacist services.

## Patients and methods

This study was a retrospective review of electronic medical record (EMR) clinical data from patients initiated on CSII therapy at a large private practice by clinical pharmacists in Northeast Tennessee. The practice is a physician-owned, primary care group consisting of 90 providers representing internal medicine, family medicine, pediatrics, obstetrics, and gynecology. The clinical pharmacists are employed with a local college of pharmacy which contracts with the practice site to provide diabetes services part-time since 2007. The pharmacists who visit with referred patients at the physician practice are certified diabetes educators (CDE) and certified pump trainers (CPT) and provide CSII services using Medtronic (Medtronic MiniMed Inc. Northridge, CA) and Animas (Johnson and Johnson Services Inc. New Brunswick, NJ) insulin pumps. Providers initiate the collaboration process by referring established patients to the pharmacist for diabetes management and education. Criteria for CSII candidacy were based on the pharmacist’s assessment of self-management skills and potential patient safety concerns.

Following an initial assessment, the pharmacists are authorized to initiate and/or intensify oral and/or injectable diabetes medication therapies, including insulin, independently via a collaborative practice agreement. This delegation of insulin management by the physician allows for dosage modification to be performed by the pharmacists including calculation of CSII starting doses and any subsequent adjustments based on downloadable insulin pump or meter reports. The CSII-based services performed by the pharmacist include, at a minimum, a 60-min initial assessment visit, 2-h CSII initiation and training visit, and at least one 30-min follow-up visit. Telephone follow-up calls were performed 24 h post-CSII initiation and as needed. CSII-based education provided by the pharmacist included basics on insulin pump therapy, acute management protocols, carbohydrate counting, insulin infusion site care, and self-monitoring blood glucose plans.

Prior to initiating the study, Institutional Review Board approval was granted by East Tennessee State University. Practice records were used to identify all patients who completed pharmacist-led CSII conversion during years 2007–2010. Inclusion criteria included patients referred by their PCP to the pharmacist for diabetes management, at least 18 years old, diagnosed with type 1 or type 2 diabetes mellitus, received previous treatment with MDI defined as the documentation of two insulins in the patient’s medication profile at the time of referral, and completed at least one diabetes-related office visit with the PCP post-CSII. Exclusion criteria included any patients previously treated with CSII who were resuming this method of insulin delivery from MDI. The pre- and post-CSII periods were defined as the 12-month periods before and after CSII initiation, respectively.

A total of 50 eligible patients were identified for the study. An informational letter explaining the study objectives/security of patient health information and a patient satisfaction survey were mailed to each eligible participant on October 1, 2011 and November 1, 2011. A follow-up phone call from the primary investigator was made to recipients within 2 days after the first mailing. A voicemail message was left if there was no answer. Recipients were encouraged to read and sign the letter, complete the patient satisfaction survey, and return it to the investigators using a pre-stamped, self-addressed envelope that served as consent for the study. The section of the survey instrument to assess participant satisfaction with CSII compared to MDI was adapted by the investigators from previous survey research conducted by Gentry *et al*.3. Survey items assessing participant satisfaction with the clinical pharmacist’s were created by the investigators. The clinical data obtained from the EMR included patient HbA1c, BMI, planned or unplanned diabetes-related PCP office visits defined as any visit with ICD-9 diabetes diagnosis documentation in the assessment/plan, and number of non-insulin diabetes medication defined as any new or continued medication prescribed at any time pre- and post-CSII.

Descriptive statistical analysis of clinical data was performed using a paired *t*-test or Wilcoxon signed rank test depending on normality analysis of the data, respectively. Survey data responses comparing CSII to MDI were analyzed using a Wilcoxon signed rank test. To assess sustainability of control, the mean HbA1c was assessed across 3-month intervals beginning at CSII initiation and continuing for 12 months post-CSII initiation. The change over time was analyzed using a one-way repeated measures analysis of variance followed by the Holm-Sidak method. Baseline characteristics for HbA1c, participant age, and BMI were reported as mean ± standard deviation. Study results for HbA1c and diabetes-related PCP visits were reported as mean ± standard error of the mean. Survey results were reported as median and interquartile range (IQR). The alpha level was set *a priori* at 0.05. Statistical analysis was performed using SigmaPlot 12.0 (Systat Software, Inc.; San Jose, CA).

## Results

**Figure 1.**
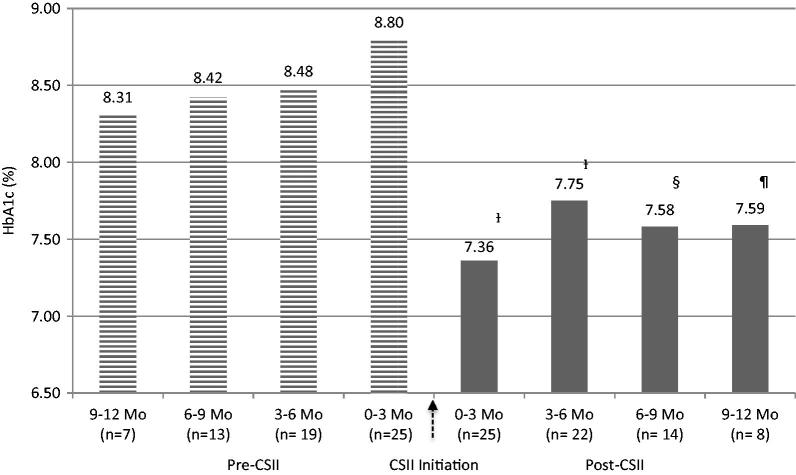
Effectiveness and sustainability of glycemic control with CSII therapy. Statistical analysis of HbA1c 0–3 months Pre-CSII vs all HbA1c intervals post-CSII. †*p* < 0.05, §*p* = 0.051, ¶*p* = 0.063. Data are represented as mean HbA1c. CSII, Continuous Subcutaneous Insulin Infusion; HbA1c, hemoglobin A1c; Mo, months.

Twenty-five patients consented to the study and returned completed surveys representing a 50% response rate. Baseline characteristics can be found in Table 1. The mean HbA1c pre-CSII was 8.69 ± 0.32% and post-CSII was 7.52 ± 0.18%, representing an absolute HbA1c reduction of −1.17% (*p* < 0.001). Figure 1 illustrates HbA1c trends over the 2-year span of pre- and post-CSII. Mean BMI post-CSII decreased by 0.7 kg/m^2^ (*p* = 0.085). Fewer diabetes-related PCP visits were completed post-CSII (5.1 ± 0.6 vs 3.8 ± 0.3 visits/year, *p* = 0.009) and less non-insulin diabetes medications were prescribed post-CSII (1.5 ± 0.3 vs 0.5 ± 0.1 medications/year, *p* < 0.001). The most prescribed non-insulin diabetes medication was metformin, with 58.9% of type 2 patients continuing therapy post-CSII. All patients treated with metformin post-CSII received it during the year before pump transition. No type 1 patients received non-insulin diabetes medications at any time before or after CSII initiation. No new non-insulin medications were prescribed for type 2 patients post-CSII, except for one patient who began an incretin-based therapy (i.e., GLP-1 agonist) 2 months after starting the insulin pump.

**Table 1.** Baseline characteristics and medications used prior ro CSII.

	*n* = 25
Male (%)	40
Female (%)	60
Age (years)	62 ± 10.14
Type 1 Diabetes Mellitus (%)	20
Type 2 Diabetes Mellitus (%)	80
HbA1c (%)	8.69 ± 1.60
Body Mass Index (kg/m^2^)	32.9 ± 8.53
Medications used prior to CSII*^a^* (%)	
Metformin	88.2
Sulfonylurea	52.9
Thiazolidinedione	29.4
Dipeptidyl peptidase 4 inhibitor	17.6
Alpha-Glucosidase Inhibitor	5.9

Baseline characteristics reported as mean ± standard deviation or as a percentage. HbA1c, Hemoglobin A1c; CSII, Continuous Subcutaneous Insulin Infusion. ^a^Baseline medication usage for only patients with diagnosis of type 2 diabetes during 1 year pre-CSII.

Survey results are summarized in Table 2. Study participants experienced greater comfort controlling glycemic excursions (e.g., high and low sugars) and making subsequent insulin dose adjustments with CSII compared to MDI (*p* < 0.001). Patients were also very confident and satisfied with clinical pharmacists’ abilities and the level of care received during their transition from MDI to CSII therapy.

**Table 2.** Satisfaction survey results.

	Response (*n* = 25)
*Insulin pump satisfaction questions*	
1. I felt in control of low blood sugars with insulin injections	2 (2.00–3.00)
2. I felt in control of high blood sugars with insulin injections	2 (2.00–3.00)
3. I feel in control of low blood sugars with my pump	4 (4.00–5.00)
4. I feel in control of high blood sugars with my pump	4 (4.00–5.00)
5. My daily activities, such as work or exercise, were affected with injections	4 (2.75–4.00)
6. My daily activities, such as work or exercise, are now affected with my pump	3 (2.00–4.00)
7. I felt comfortable making dosage changes to my insulin with injections	2 (2.00–4.00)
8. I feel comfortable making dosage changes to my insulin with my pump	4 (4.00–5.00)
9. Overall, I find my insulin pump to be more convenient than injections	5 (5.00–5.00)
10. Overall, I prefer my insulin pump over injections	5 (5.00–5.00)
11. I would recommend an insulin pump to others	5 (5.00–5.00)
*Pharmacist satisfaction survey questions*	
12. I felt confident in the ability of the pharmacist who trained me on my pump	5 (5.00–5.00)
13. I feel confident in the ability of the pharmacist adjusting my pump settings	5 (5.00–5.00)
14. I felt confident in the pharmacist’s knowledge of pump therapy	5 (5.00–5.00)
15. I would recommend working with a pharmacist to others considering a pump	5 (4.75–5.00)
16. I feel the expertise of pharmacists with medications and medication-related devices (e.g., insulin pumps) make them an ideal provider to work with my physician when starting an insulin pump	5 (5.00–5.00)

Results reported as median (interquartile range); 1 = Strongly Disagree, 2 = Disagree, 3 = Neutral, 4 = Agree, 5 = Strongly Agree.

## Discussion

CSII, as an alternative to MDI, has been employed in the treatment of type 1 and type 2 diabetes for several years. The successful transition takes a substantial amount of face-to-face time and requires the assistance of clinicians skilled in the knowledge of CSII therapy, insulin management, and nutritional education (e.g., carbohydrate counting). Several studies have previously evaluated the use of CSII in patients with diabetes2–9,22. Outcomes from most investigations directly comparing CSII vs MDI have demonstrated an overall reduction in HbA1c with CSII between the two methods6,8,22. Outcomes from transitional studies include improved glycemic control after switching from MDI to CSII3–5,7. Further results from studies demonstrate that, while CSII therapy has improved glycemic control, there has not been a causative increase in minor or severe hypoglycemia events which is an important patient safety concern4,6. Finally, CSII-based therapy has resulted in a reduction in albumin excretion rates22 and improved patient quality-of-life9.

The results from our study demonstrate a significant reduction in the overall mean HbA1c 1-year post-CSII. This finding was consistent with other studies comparing baseline HbA1c to 12 months post-CSII initiation4,5,7. Of note, our baseline mean HbA1c pre-CSII was lower (8.69%) compared to other baseline HbA1c values from transitional studies3,5,7, thus the absolute mean HbA1c reduction of −1.17% was impressive. We also stratified HbA1c across 3-month intervals post-CSII over 12 months to assess the sustainability of glycemic control following conversion. Significantly improved HbA1c was observed for the first 6 months, with results for the last 6 months trending toward significance (see Figure 1). A possible explanation for the insignificant results is likely due to the study’s small sample size. Although statistically insignificant, this level of glycemic improvement still carries clinical significance.

Results from previous CSII-based trials have exhibited varying effects on weight or BMI3,6–8. Improved diabetes control using insulin intensification typically results in weight gain. The results from our study revealed a small, but insignificant reduction in BMI. This reduction in BMI may encourage patients to consider CSII who are reluctant to intensify their insulin therapy due to fear of weight gain. To our knowledge, the current study was the first to measure and compare diabetes-related PCP visits pre- and post-CSII. This outcome was important in order to demonstrate that working collaboratively with the clinical pharmacist does not completely disband the patient–physician relationship. Rather it more accurately demonstrated a workload shift since the pharmacists were located within the physician practice. The decision to analyze only PCP visits with an ICD-9 diagnosis code for diabetes and not all PCP visits over the study period was to eliminate any potential for exaggerating the results based on office encounters where the physician’s documented clinical decision-making was not related to diabetes. The results indicate that patients visited their PCP ∼4-times per year after transition, which is consistent with an appropriate diabetes follow-up plan in the primary care setting. As expected, CSII initiation resulted in fewer non-insulin diabetes medications prescribed in type 2 patients, which highlights the effectiveness of this insulin intensification approach. Most type 2 patients (58.9%) continued metformin therapy and one patient (5.9%) began GLP-1 agonist 2 months post-CSII for weight control. However, the GLP-1 agonist was only used for less than 4 months in this patient. While GLP-1 agonists have the potential to influence glycemic control and body weight, the limited exposure in this occurrence was not likely to affect the study results significantly.

The survey assessed patient satisfaction with CSII compared to MDI and satisfaction with the pharmacist-led CSII services. Assessing patient satisfaction with the clinical pharmacist is a qualitative outcome unique to our study. Previous studies have assessed patient satisfaction with CSII compared to MDI, with results showing that patients not only prefer CSII, but there is an improvement in patient quality-of-life3,9. Consistent with results from Gentry *et al*.3, patients felt that they were more in control of hyperglycemia and hypoglycemia with CSII. They also felt more comfortable making insulin dose changes using CSII compared to MDI. Patients were very confident in the ability of the clinical pharmacist to train them on CSII therapy and adjust insulin pump settings based on self-monitoring of blood glucose (SMBG) patterns. Patients also felt the pharmacist was knowledgeable regarding CSII therapy and would recommend working with a pharmacist–physician team to other patients considering this method of insulin delivery. While our satisfaction survey focused only on the abilities of clinical pharmacists, patients may have reported similar scores with other health practitioners who assist in CSII initiation.

This study has several limitations. First, it analyzed a small patient population at one practice site without the presence of a comparator group. Since all CSII initiations at this practice location are initiated and managed by clinical pharmacists, it was not possible to compare the study’s outcomes with a group where CSII was initiated and managed by other healthcare providers. Second, several confounding factors were not accounted for and may have contributed to the outcomes. For example, dietary adherence (e.g., carbohydrate counting) or patient compliance with self-monitoring of blood glucose (SMBG) were not measured pre- and post-CSII. Initiating or intensifying these skills can influence diabetes control in patients employing either MDI or CSII-based therapies. Also, the intensification in health provider contact via office visits and telephone follow-up that is required for CSII initiation and adjustments may have influenced results and not solely the CSII *per se*. Plus, while the majority of type 2 patients stopped all non-insulin diabetes medications with the lone exception of metformin, one patient began a GLP-1 agonist, albeit only for a short period of time. Third, the study used a patient satisfaction survey that was partially created by the investigators and has not been validated. Finally, specific types of biases may have prejudiced survey results. For example, a low response rate of 50% may represent a voluntary bias indicating opinions only from patients who had very positive experiences with CSII transition. An acquiescence bias may have been present too, as patients may feel they are unable to report negatively to the investigators about their experiences if they must also maintain that relationship for future CSII-related needs.

In conclusion, CSII initiated and managed by pharmacists working under a collaborative care agreement is associated with improved glycemic control following conversion from MDI without a significant increase in BMI. The collaboration also resulted in a decrease in diabetes-related PCP visits over 12 months following CSII initiation, however patients were still seen at recommended intervals (e.g., every 3–4 months). Patients appear to be very satisfied with this form of insulin delivery and with the clinical pharmacists’ abilities to initiate and manage CSII therapy. Physician–pharmacist collaboration to provide CSII services appear promising and this study should only serve as a preliminary confirmation that this partnership can be beneficial in primary care settings without an endocrinologist. Future investigations are warranted and should involve multiple centers involving a larger number of patients, include patient safety outcomes (e.g., hypoglycemia, hospitalizations), and capture qualitative data from referring physicians.

## Transparency

### Declaration of funding

The authors received no payment in preparation of this manuscript. The Bill Gatton College of Pharmacy paid for the mailing of the surveys. The insulin prescription and insulin pumps were purchased by patients (through insurance) and not by the clinic or funded through a third party.

### Declaration of financial/other relationships

RH received compensation from Medtronic for instruction and training of patients on personal continuous glucose monitoring (CGM system). The other authors declare no conflicts of interest.

## Acknowledgments

The authors wish to thank Ralph Lugo, PharmD, for assistance with statistical analysis and Nick Hagemeier, PharmD, PhD, for editorial comments.
